# ﻿A new species of genus *Chiloschista* (Aeridinae, Vandeae, Epidendroideae, Orchidaceae) from Sumatra Island, Indonesia

**DOI:** 10.3897/phytokeys.252.138190

**Published:** 2025-02-12

**Authors:** Destario Metusala

**Affiliations:** 1 Research Center for Biosystematics and Evolution, National Research and Innovation Agency (BRIN), Jl. Raya Jakarta-Bogor km. 46, Cibinong, West Java, Indonesia National Research and Innovation Agency (BRIN) Cibinong Indonesia; 2 Purwodadi Botanic Garden, National Research and Innovation Agency (BRIN) Jl. Raya Surabaya-Malang km.65, Pasuruan, East Java, Indonesia National Research and Innovation Agency (BRIN) Cibinong Indonesia

**Keywords:** Aceh, leafless orchid, morphology, Southeast Asia

## Abstract

*Chiloschistatjiasmantoi*, a new species of epiphytic leafless orchid from the northernmost region of Sumatra Island, Indonesia, is described and illustrated. The flower of this new species is morphologically close to *C.javanica*, but differs in having oblong-obovate petals, narrowly oblique oblong side lobes with truncate to obtuse apex, and a different shape of lip sac.

## ﻿Introduction

The Indonesian archipelago is known to be one of the most important orchid diversity hotspots in the world, and it continues to generate new species discoveries, which indicates that there are still many potential areas that need to be explored (e.g., Metusala 2017a; Metusala and Supriatna 2017; Metusala 2019a, 2019b; Metusala and O’Byrne 2020; Saputra et al. 2020; Metusala et al. 2021; Metusala 2024). Recent botanical explorations in Indonesia have usually focused on a few popular, orchid taxa, while groups such as mycoheterotropic and leafless orchids have been comparatively neglected, presumably due to their less attractive habitus, often cryptic growth habit and small flower size (Metusala and Supriatna 2017; Metusala 2020a; Suetsugu et al. 2021; Panday et al. 2022).

The leafless orchid genus *Chiloschista* Lindl. (1832: 1522) was established in 1832 with *C.usneoides* (D.Don) Lindl. (1832: 1522) as its type, and there are now 30 accepted species distributed from the Indian subcontinent through to Southeast Asia and Australia (Dalstrom and Kolanowska 2020; POWO 2024). In previous treatments, the genus was classified under various subtribes, such as Sarcochilinae (Schlechter 1926), Aeridinae (Dressler 1993), and Phalaenopsidinae (Szlachetko 2003). However, recent phylogenetic studies showed that *Chiloschista* is a monophyletic genus, clustered with *Phalaenopsis*, within the subtribe Aeridinae of the tribe Vandeae (Zou et al. 2015; Liu et al. 2023).

Species of this genus are characterized by monopodial growth, typically as epiphytic or lithophytic herbs. Plants are often seen as a cluster of numerous terete or flattened photosynthetic roots that radiate from a very short stem. *Chiloschista* flowers are either ephemeral or last up to several days, 3-lobed, saccate or spurred, and usually have a thickened hairy internal callus. Their columns are subterete and short, but have a rather long foot. The anther caps have 2 filiform setae and 4 pollinia in 2 closely appressed pairs (Cockburn et al. 1985; Chen and Wood 2009; Wood 2014; Dalstrom and Kolanowska 2020).

Prior to this article, Indonesia has 4 accepted species: *C.javanica* Schltr. (1919: 275) is recorded only from Java Island; *C.phyllorhiza* (F.Muell) Schltr. (1921: 492) is distributed across Java, the Lesser Sunda Islands, and Sulawesi; *C.taeniophyllum* (J.J.Sm.) Schltr. (1921: 492) is endemic to Maluku (Ambon Island and Banda Islands); and *C.treubii* (J.J.Sm.) Schltr. (1921: 492) that is also endemic to Maluku (Seram Island and Wokam Island of the Aru Islands Regency) (POWO 2024). There is no prior record of any *Chiloschista* species from Sumatra, Borneo, and New Guinea Islands.

During a botanical inventory conducted by the author in 2019, several living specimens of *Chiloschista* were found growing on semi-open coffee plantation close to the forest in Aceh Province, in the northernmost region of Sumatra Island. *Chiloschista* roots usually turn darker when wet and look like the color of tree bark, which makes them difficult to find, such that their small but bright flowers are often important to their detection. Several plants, including individuals with flowers, were collected as herbarium and living specimens for Purwodadi Botanic Gardens in East Java. Further observations of the flowering specimens found that this taxon represents an undescribed species with flower characteristics morphologically similar to *C.javanica* and *C.sweelimii*. Here I describe it as a new species, as well as the first record of this genus on Sumatra Island. A key to the five species of *Chiloschista* in Indonesia is also included.

## ﻿Materials and methods

Morphological measurements were conducted with a loupe and a ruler accurate to 0.5 mm. Environmental data were collected using thermohygrometer and lux–meter. The specimens were also compared with closely related species (*C.javanica* and *C.sweelimii*). Observation and morphological studies have been done by examining the relevant literature sources, illustrations, living and herbarium specimens, and photographs. These literature sources included protologues and descriptions of the relevant taxa: *C.javanica* (Smith 1905a), *C.sweelimii* (Teck 2016), *C.taeniophyllum* (Smith 1905b), *C.treubii* (Smith 1912), and *C.phyllorhiza* (Mueller 1866; Smith 1939). Photographs were taken with a Sony DSC-W70. Detailed morphological observations were conducted using stereomicroscope Olympus SZX7 with camera EP50. Terminology for morphological description follows Harris and Harris (2001). Conservation status was assessed using the IUCN Red List Category and Criteria. The Extent of Occurrence (EOO) and Area of Occupancy (AOO) were estimated based on GeoCAT (https://geocat.iucnredlist.org/). Six specimens of *Chiloschista* sp. from Aceh were used for examinations (*RIO 9117*; *9118*; *9119*; *9121*; *9123*; *9124*).

### ﻿Additional examined specimens

1. *Chiloschistasweelimii*. Malaysia • Malay Peninsula; *Lim*, *S.L. s.n*.; K 000891272 • Malay Peninsula; *Ong FRI 75458* (KEP).

2. *Chiloschistajavanica*. Indonesia • Java; *Docters van Leeuwen s.n.*, L 1500077 • Java; *leg. ign. s.n*.; L 0264545 • Java; *Docters van Leeuwen 2402*; BO 0057026 • Java; *Docters van Leeuwen 2402*; BO 0057027 • Java; *leg. ign. s.n.*; BO 0057030 • Java; Docters *van Leeuwen s.n.*; BO 0057031 • Java; *Docters van Leeuwen s.n.*; BO 0057037 • Java; *leg. ign. s.n*.; BO 0057038.

## ﻿Taxonomic treatment

### ﻿ Chiloschista tjiasmantoi

Taxon classificationPlantae AsparagalesOrchidaceae

Metusala sp. nov.

F384924D-3987-587C-9228-9507231E449E

urn:lsid:ipni.org:names:77356634-1

**Figure 1.**
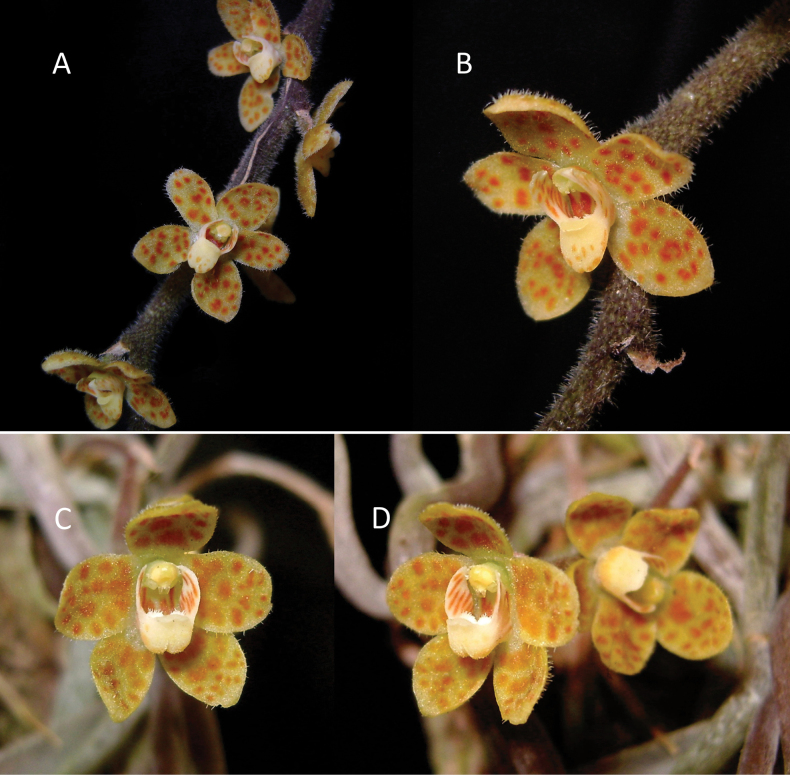
*Chiloschistatjiasmantoi* sp. nov. (**A, B**) and *Chiloschistajavanica* (**C, D**) **A** inflorescence **B** flower, oblique view. **C** flower, front view **D** flower, oblique view. Photos by Destario Metusala.

**Figure 2.**
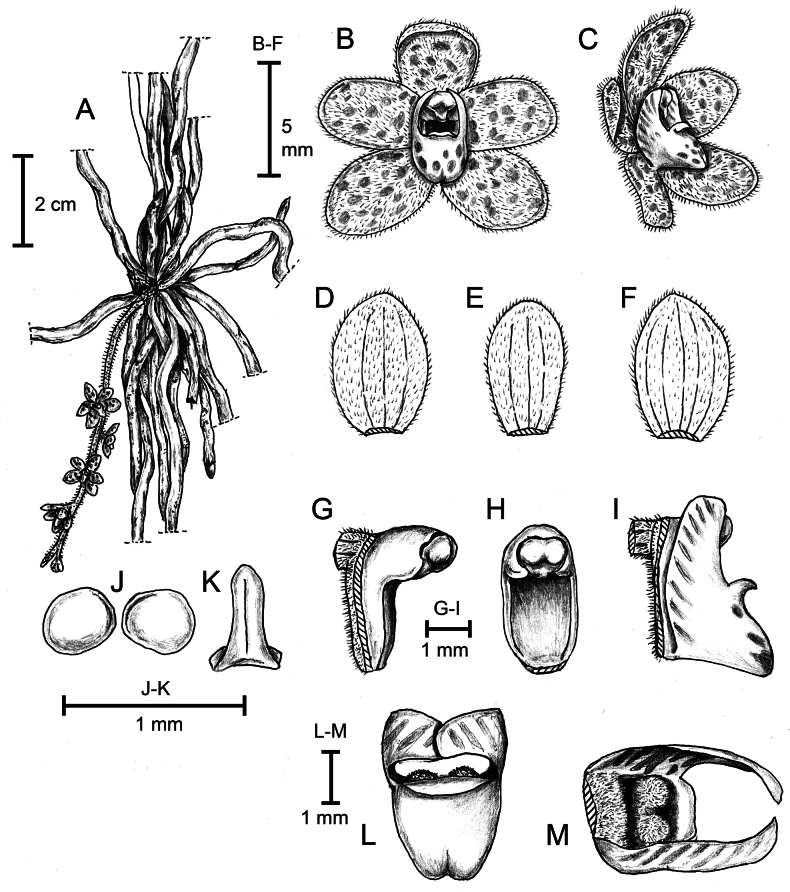
*Chiloschistatjiasmantoi* sp. nov. **A** habitus with inflorescence **B** flower, natural shape, front view **C** flower, natural shape, oblique view **D** dorsal sepal, flat shape **E** petal, flat shape **F** lateral sepal, flat shape **G** column and foot, oblique view **H** column and foot, front view **I** column and lip, side view **J** pollinia **K** stipe and viscidium **L** lip, above view **M** lip interior, back view. Line drawing by Destario Metusala from *RIO 9118*.

**Figure 3.**
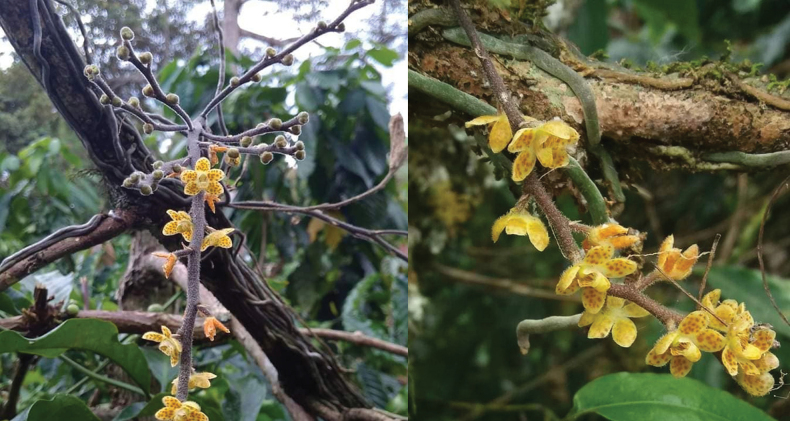
*Chiloschistatjiasmantoi* sp. nov. Flowering plants *in situ*. Photos by Alfajaruddin.

**Figure 4.**
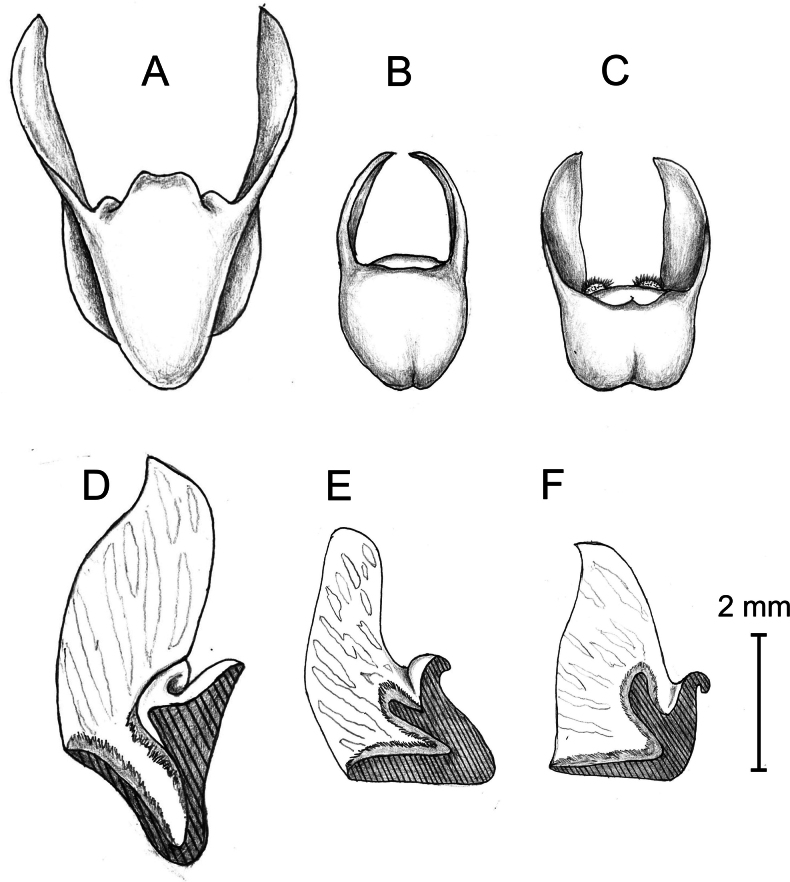
Lip of three *Chiloschista* species **A***C.sweelimii*, natural shape, front view **B***C.tjiasmantoi*, natural shape, front view **C***C.javanica*, natural shape, front view **D***C.sweelimii*, longitudinal section, flat shape, side view **E***C.tjiasmantoi*, longitudinal section, flat shape, side view **F***C.javanica*, longitudinal section, flat shape, side view. **A** and **D** redrawn after Ong Poh Teck in Teck (2016). Drawn by Destario Metusala.

Figs 1A, B, 2, 3, 4B, E

#### Type.

Indonesia • Sumatra: Aceh Province, c. 900 m, *RIO 9118* (holotype, BO!) (detailed localities are not shown here for conservation purpose).

#### Diagnosis.

*Chiloschistatjiasmantoi* is morphologically similar to *C.javanica*, but differs in having oblong-obovate petals (vs. broadly elliptic to ovate petals), narrowly oblique oblong side lobes with truncate to obtuse apex (vs. relatively straight triangular side lobes with obtuse apex), a lip sac that has a “V” shape in longitudinal section view with a narrow angle of about 45–50° (vs. a lip sac that has an “L” shape in longitudinal section view with a wide angle of about 90°), and a very narrow cavity between the apex of the hairy callus and the thick curved front lobe of the lip (vs. a rather broad cavity).

#### Description.

Epiphytic herb. Roots numerous, spreading, terete to slightly flattened, 2.0–3.5 mm in diameter, greyish-green when wet and becoming grayish-white when dry, grow radially from a short stem as the central, and mature individuals can grow elongated to reach more than 30.0 cm. Stem reduced, very short, erect, simple 2.0–4.0 mm long, up to 3.0 mm in diameter, densely covered with dry stem bracts. Stem bracts are triangular active, and persistent, and encircle the stem tightly. Leaves one or two, 4–7 mm long, deciduous, unseen in cultivation. Inflorescence axillary, arising among the roots gap, pendulous, up to 31.0 cm long in total, peduncle c. 2.0 mm in diam. near base, terete, densely covered with short white hairs, purplish near base and becoming purplish green toward apex, sometimes branched at the base, up to 30 flowered per rachis, flowers arranged spirally in a slightly zig-zag pattern and open simultaneously, each flower can last up to 5 days; flower bracts triangular, 2.0–3.0 mm long × 1.5–2.0 mm wide, acuminate to caudate, pubescent, greenish and soon becoming brown when old. Pedicel and ovary about 2.0 mm long, terete, brownish-green to purplish-green, covered with whitish hairs. Flower rather thin-textured, 1.0–1.2 cm high × 1.0–1.2 cm wide, open widely, sepals and petals yellowish cream or yellow with orange or reddish spots, labellum yellowish cream with reddish or orange spots on their sac, column yellowish green with orange tinge on its foot. Dorsal sepal oblong-elliptic, 5.0–6.0 mm long × 4.0 mm wide, obtuse to rounded, both surfaces pubescent. Petals oblong-obovate, 5.0–6.0 mm long × 3.0–4.0 mm wide, truncate to rounded, both surfaces pubescent. Lateral sepals obliquely oblong-elliptic, 6.0 mm long × 4.0–5.0 mm wide, obtuse, both surfaces pubescent. Lip immobile, minutely papillose externally, 3-lobed, deeply saccate, indistinctly canaliculated ventrally, 2.8–3.2 mm long, 4.0–4.5 mm high (from side lobes apex to basal part of sac), 2.0–2.5 mm wide at front; side lobes erect to slightly curved inwards, obliquely oblong to rather falcate, c. 2.0 mm long × c. 1.5 mm wide near base and narrowed gradually to about 1.0 mm near apex, apex truncate to rounded, yellowish cream with red streaks on internal surface; front lobe subtrapezoid, short c. 1.0–1.5 mm × 1.5 mm, apex truncate to slightly emarginate, curved; sac ovate to subrectangular from front view, “V” shape in longitudinal section view, 2.0–2.5 mm long × 2.0–2.3 mm wide, apex truncate or retuse or slightly bilobed, yellowish cream with pale reddish or pale orange spots on external surface around the apex; callus a fleshy thickening arise from basal to middle of the internal front wall, split into two oblong hairy callus that rises up to sac opening, creating a small narrow cavity between apex of the hairy callus and the thick curved front lobe of the lip; column short 1.5–2.0 mm long (excluding the anther cap), foot about 2.2–2.5 mm long; anther cap cucullate, yellowish or cream, c. 1.5 mm × 1.5 mm; pollinarium two unequal globose on a narrowly linear to triangular stipe, yellow. Fruit not seen.

#### Distribution, habitat and phenology.

Based on the existing data, the distribution of *Chiloschistatjiasmantoi* may be restricted to Aceh Province, the most northern part of Sumatra Island. This new species is currently only known from five locations in two different regencies at elevation ranging from 700–1000 m. The populations of this species were mostly found growing epiphytically on old coffee trees (*Coffea* spp.) and shade trees (*Leucaena* spp.) in the local coffee plantations, together with *Vandapumila* (Orchidaceae), in a windy and semi–opened wet habitat with medium sunlight intensity. Flowering recorded in mid-July, early November to late December.

#### Etymology.

The specific epithet “tjiasmantoi” honors Wewin Tjiasmanto, the chairman of Tjiasmanto Conservation Fund and a philanthropist concerned with the Indonesian plant conservation.

#### Cultivation.

*Chiloschistatjiasmantoi* seems rather difficult to cultivate at lower elevations (300 m. a.s.l). However, it was successfully grown by attaching it to a slab of tree fern with a top dressing of moss to prevent the roots from drying out, under a light intensity of about 30–75% with good air circulation and humidity levels of about 80% or more.

#### Discussion.

This new species is morphologically similar to *C.javanica* (Fig. 1C, D, 4C, F) and *C.sweelimii* (Fig. 4A, D). However, there is a geographical separation among these three species. *C.tjiasmantoi* is thus far only recorded from Aceh Province, the northernmost region of Sumatra Island; *C.sweelimii* occurs in the Malay Peninsula and Vietnam, and *C.javanica* is endemic to Java Island.

*Chiloschistatjiasmantoi* differs from *C.javanica* in having narrower oblong-obovate petals, narrowly oblique oblong side lobes with truncate to obtuse apex, a lip sac that has a “V” shape in longitudinal section view with a narrow angle of about 45–50°, a narrow cavity between the apex of the hairy callus and the thick curved front lobe of the lip. Meanwhile, *C.javanica* has broadly elliptic to ovate petals, relatively straight triangular side lobes with obtuse apex, a lip sac that has an “L” shape in longitudinal section view with a wide angle of about 90°, and a rather broad cavity between the apex of the hairy callus and the thick curved front lobe of the lip.

Furthermore, *C.tjiasmantoi* differs from *C.sweelimii* in having sepals and petals with hairs on both sides, oblong-obovate petals, a broadly oblong-elliptic dorsal sepal, basal front lobe without any callus, an ovoid to subrectangular lip’s sac from front view with a truncate to slightly bilobed apex, and an oblong fleshy thickening that arises from basal to middle of the internal front wall. This thickening then divides into a 2-lobed hairy callus that rises near the sac opening, creating a cavity between the apex of the hairy callus and the thick curved front lobe. In contrast, *C.sweelimii* has sepals and petals with hairs only on their adaxial surface, ovate to orbicular petals, an ovate dorsal sepal, basal front lobe with large callus on either side (each c. 1.5 × 1.0 mm), a triangular shaped lip’s sac from front view with pointed obtuse apex, and the absence of prominent internal callus inside the lip’s sac (Teck 2016). A comparison between the new species and its morphological allies is shown in Table 1.

**Table 1.** Morphological comparison among the new species and its allies.

Characters	*C.tjiasmantoi* Metusala	*C.javanica* Schltr	*C.sweelimii* Holttum
Flower colour	Sepals and petals yellowish cream or yellow with orange or reddish spots, lip yellowish cream with reddish or orange spots on their sac and side lobes with pale reddish orange stripes	Sepals and petals yellowish cream or yellow with orange or reddish spots, lip white or cream with pale reddish or orange spots on their sac and side lobes with pale reddish orange stripes	Sepals and petals yellow with orange-brown blotches, lip white on their sac and side lobes with reddish-orange stripes
Dorsal sepal	Oblong-elliptic, 5.0–6.0 mm long × 4.0 mm wide, obtuse to rounded, both surfaces pubescent but abaxial with shorter hairs	Elliptic, 5.0–6.0 × 3.0–3.5 mm, obtuse to rounded, adaxial surface pubescent, abaxial with scattered shorter hairs	Ovate, 5.0–6.0 × 3.5–5.0 mm, obtuse to acute, only adaxial surface pubescent
Lateral sepals	Obliquely oblong-elliptic, 6.0 mm long × 4.0–5.0 mm, obtuse, both surfaces pubescent but abaxial with shorter hairs	Elliptic, 4.0–5.0 mm × 3.0–3.5 mm, obtuse to rounded, adaxial surface pubescent, abaxial with scattered shorter hairs	Broadly elliptic to broadly ovate, 5.0–6.0 × 3.5–5.0 mm, obtuse to acute, only adaxial surface pubescent
Petals	Oblong-obovate, 5.0–6.0 mm long × 3.0–4.0 mm wide, truncate to rounded, both surfaces pubescent but abaxial with shorter hairs	Broadly elliptic to ovate, 4.0–4.5 × 3.0–3.5 mm, obtuse to rounded, adaxial surface pubescent, abaxial with scattered shorter hairs	Ovate-orbicular, 4.0–5.0 × 3.0–4.5, obtuse, adaxial surface sparsely pubescent
Side lobes	Obliquely oblong, slightly falcate, *c*. 1.5 mm wide at base and narrowed gradually to about 1.0 mm near apex, truncate to obtuse	Triangular, relatively straight, *c*. 1.9 mm wide at base and narrowed gradually to about 0.8 mm near apex, acute	Obliquely oblong, slightly falcate, c. 2 mm wide at base and narrowed gradually to about 0.9 mm near apex, acute to obtuse
Front lobe	Broadly subtrapezoid, short, *c*. 1.0–1.5 × 1.5 mm, apex truncate to slightly emarginate, curved, base without any callus	Broadly subtrapezoid, short, *c*. 1.0–1.5 × 1.5 mm, apex truncate to slightly emarginate, curved, base without any callus	Broadly triangular, short, *c*. 2.0 × 1.0 mm, apex retuse, base with large callus on either side (*c*. 1.5 × 1.0 mm)
Lip’s sac	Ovate to subrectangular from front view, forming a “V” shape in longitudinal section view with a narrower angle (45–50°), *c*. 2.0–2.5 × 2.0–2.3 mm, apex retuse to slightly bilobed	Subrectangular from front view, forming an “L” shape in longitudinal section view with a wider angle (90°), *c*. 2.0–2.3 × 2.7–3.0 mm, apex retuse	Triangular from front view, forming a “V” shape in longitudinal section view with a narrower angle (45–50°), *c*. 4.0–5.0 × 3.0–3.5 mm, apex obtuse
Internal lip’s sac ornament	An oblong fleshy thickening arises from basal to middle of the internal front wall, split into 2 lobed hairy callus that rises to sac opening, creating a small narrow cavity between apex of the hairy callus and the thick curved front lobe	A large and high oblong fleshy thickening arises from basal to middle of the internal front wall, split into 2 lobed long protruding hairy callus that rises to sac opening, creating a cavity between apex of the hairy callus and the thick curved front lobe	Internal callus absence

This new species will be the fifth *Chiloschista* species in Indonesia, and the first record of this genus on Sumatra Island. With the exception of *C.phyllorhiza*, the species are endemic to the country and appear to have restricted distributions. Although the flowers of some species appear nearly identical in appearance, the internal structure of the lip can be very different, which suggests this would be a good key character for further identification of this genus (Gyeltshen et al. 2019; Dalstrom and Kolanowska 2020). A deeper investigation into its floral morphological variation needs to be carried out in the future, as this is essential to support species delimitation of Indonesian *Chiloschista* (Metusala 2020b; Metusala et al. 2020).

#### Conservation.

*Chiloschistatjiasmantoi* was recorded from five locations in Aceh Province. Based on currently available data, the Extent of Occurrence (EOO) of this species is 117.01 km^2^ with user-defined cell width = 2 km (criterion B1: < 5,000 km^2^) and Area of Occupancy (AOO) value of 20.00 km^2^ (criterion B2: < 500 km^2^). Their natural habitat is threatened by land conversion, especially from large-scale coffee plantations. Existing populations were estimated to be less than 2,500 mature individuals (≤ 250 in most subpopulations) where they mostly found attached to the branches of coffee trees and its shade trees (*Leucaena* spp.). Unfortunately, coffee plantations provide a vulnerable habitat for this new orchid species as they can be pruned or cut down at any time. Moreover, many coffee farmers believe that this orchid is a harmful parasitic plant and eradicate them by clearing the coffee branches from any epiphytic plants. This species has also been found traded on domestic online platforms as an ornamental orchid, although currently in small quantities as its habit and small flowers are considered less attractive to Indonesian hobbyists. In many cases, unsustainable commercial harvesting may have a significant impact on the viability of wild orchid populations (Hinsley et al. 2018). Therefore, I consider this species to likely be in the Endangered category B1 and B2+ab (i,ii,iii,iv), C2+a (i) (IUCN Red List Categories and Criteria).

It has been well-documented that ecosystems in the highland of Aceh Province are severely affected by climate change, particularly through increased temperatures (Schroth et al. 2015). This could also have a major impact on various sensitive plant species, such as many highland epiphytic orchids, including natural populations of *Chiloschistatjiasmantoi*. Therefore, conservation-based researches are needed to determine the vulnerability of these species to climate change-related pressures, especially drought stress (Al Farishy et al. 2017; Arimy et al. 2017; Metusala 2017b; Metusala et al. 2017; Suffan et al. 2021; Ishmah et al. 2021; Trimanto et al. 2023).

### ﻿Key to the species of *Chiloschista* in Indonesia

**Table:** 

1	Roots very flattened with a glabrous inflorescence’s peduncle	**2**
–	Roots more or less terete with a hairy inflorescence’s peduncle	**4**
2	Sepals and petals narrowly elliptic, the lip’s sac conical with pointed apex	***C.taeniophyllum***
–	Sepals and petals broadly elliptic, the lip’s sac hemispherical with rounded or slightly bilobed apex	**3**
3	Lip’s sidelobes obliquely quadrangular with sac apex rounded	***C.treubii***
–	Lip’s sidelobes oblong with sac apex slightly bilobed	***C.phyllorhiza***
4	Lip’s sac has a “V” shape in longitudinal section view with a narrow angle of about 45–50°	***C.tjiasmantoi***
–	Lip’s sac has an “L” shape in longitudinal section view with a wide angle of about 90°	***C.javanica***

## Supplementary Material

XML Treatment for Chiloschista tjiasmantoi

## ﻿Additional information

### Author ORCIDs

Destario Metusala https://orcid.org/0000-0003-0483-1883

### Data availability

All of the data that support the findings of this study are available in the main text.

## References

[B1] Al Farishy DD Nisyawati Metusala D (2017) Characterization anatomical leaf blade five species *Nepenthes* from Kerinci Seblat National Park, Kerinci regency, Jambi Province. In: Mart T, Triyono D, Sugeng KA (Eds) AIP Conference Proceedings 1862, 030115. 10.1063/1.4991219

[B2] Arimy NQ Nisyawati Metusala D (2017) Comparison of leaf anatomy on some *Nepenthes* spp. (Nepenthaceae) from highland and lowland habitat in Indonesia. In: Mart T, Triyono D, Sugeng KA (Eds) AIP Conference Proceedings 1862, 030111. 10.1063/1.4991215

[B3] Chen X Wood JJ (2009) *Chiloschista*. Flora of China 25: 470–471. http://flora.huh.harvard.edu/

[B4] Cockburn W Goh CJ Avadhani PN (1985) Photosynthetic carbon assimilation in a shootless orchid, *Chiloschistausneoides* (DON) LDL. Plant Physiology 77(1): 83–86. 10.1104/pp.77.1.83 16664034 PMC1064461

[B5] Dalstrom S Kolanowska M (2020) A new yellow-flowered *Chiloschista* (Orchidaceae: Aeridinae) from Thailand. Lankesteriana 20(2): 241–248. 10.15517/lank.v20i2.43454

[B6] Dressler RL (1993) Phylogeny and classification of the orchid family. Dioscorides Press, 1–330.

[B7] Gyeltshen C Dalstrom S Gyeltshen N Tobgay K (2019) A new spotted *Chiloschista* (Orchidaceae: Aeridinae) From Bhutan. Lankesteriana 19(1): 23–29. 10.15517/lank.v19i1.37030

[B8] Harris JG Harris MW (2001) Plant identification terminology: an illustrated glossary. Spring Lake Publication, 1–206.

[B9] Hinsley A de Boer HJ Fay MF Gale SW Gardiner LM Gunasekara RS Kumar P Masters S Metusala D Roberts DL Veldman S Wong S Phelps J (2018) A review of the trade in orchids and its implications for conservation. Botanical Journal of the Linnean Society 4(186): 435–455. 10.1093/botlinnean/box083

[B10] Ishmah S Metusala D Nisyawati Supriatna J (2021) Anatomical Comparison of Two *Grammatophyllum* spp. (Orchidaceae) Species and Their Specific Ecological Adaptation. In: Herdiansyah H et al. (Eds) IOP Conference Series. Earth and Environmental Science 940: 012016. 10.1088/1755-1315/940/1/012016

[B11] Liu DK Zhou CY Tu XD Zhao Z Chen JL Gao XY Xu SW Zeng MY Ma L Ahmad S Li MH Lan S Liu ZJ (2023) Comparative and phylogenetic analysis of *Chiloschista* (Orchidaceae) species and DNA barcoding investigation based on plastid genomes. BMC Genomics 24(1): 749. 10.1186/s12864-023-09847-8 38057701 PMC10702055

[B12] Metusala D (2017a) Two new species of *Paphiopedilum* (Orchidaceae: Cypripedioideae) section Barbata from Sumatra, Indonesia. Edinburgh Journal of Botany 74(2): 169–178. 10.1017/S0960428617000063

[B13] Metusala D (2017b) An alternative simple method for preparing and preserving cross-section of leaves and roots in herbaceous plants: Case study in Orchidaceae. In: Mart T, Triyono D, Sugeng KA (Eds). AIP Conference Proceedings 1862: 030113. 10.1063/1.4991217

[B14] Metusala D (2019a) *Dendrobiumnagataksaka* (Orchidaceae: Epidendroideae), a new species of section Spatulata from Papua, Indonesia. Phytotaxa 415(5): 271–278. 10.11646/phytotaxa.415.5.3

[B15] Metusala D (2019b) A new name for an overlooked species of *Eulophia* (Orchidaceae) from Wallacea. Phytotaxa 415(4): 217–224. 10.11646/phytotaxa.415.4.6

[B16] Metusala D (2020a) *Gastrodiakhangii*, a new synonym and new record of *Gastrodiabambu* (Orchidaceae) in Vietnam. Phytotaxa 454(1): 055–062. 10.11646/phytotaxa.454.1.5

[B17] Metusala D (2020b) A new synonym of *Spathoglottistricallosa* (Orchidaceae). Phytotaxa 452(4): 298–300. 10.11646/phytotaxa.452.4.6

[B18] Metusala D (2024) A new species of *Aerides* (Aeridinae: Orchidaceae) From Sulawesi, Indonesia. Edinburgh Journal of Botany 81(2001): 1–8. 10.24823/ejb.2024.2001

[B19] Metusala D O’Byrne P (2020) *Dendrobiumrubrostriatum*, a new species of DendrobiumsectionAporum from West Kalimantan, Indonesian Borneo. Phytotaxa 443(3): 279–286. 10.11646/phytotaxa.443.3.4

[B20] Metusala D Supriatna J (2017) *Gastrodiabambu* (Orchidaceae: Epidendroideae), a new species from Java, Indonesia. Phytotaxa 317(3): 211–218. 10.11646/phytotaxa.317.3.5

[B21] Metusala D Supriatna J Nisyawati Sopandie D (2017) Comparative leaf and root anatomy of two *Dendrobium* Species (Orchidaceae) from different habitat in relation to their potential adaptation to drought. In: Mart T, Triyono D, Sugeng KA (Eds). AIP Conference Proceedings 1862: 030118. 10.1063/1.4991222

[B22] Metusala D Wibowo ARU Mambrasar YM Hendrian H (2020) A new synonym of *Bulbophyllumovalifolium* (Orchidaceae: Epidendroideae). Phytotaxa 464(3): 227–235. 10.11646/phytotaxa.464.3.4

[B23] Metusala D Saputra R Trimanto Nisyawati (2021) A new species of DendrobiumsectionSpatulata from Maluku, Indonesia. Phytotaxa 528(5): 269–278. 10.11646/phytotaxa.528.5.1

[B24] Mueller F (1866) Fragmenta Phytographiae Australiae V. Auctoritate Gubernatori Coloniae Victoriae Ex Officina Joannis Ferres, 1–252.

[B25] Panday S Agrawala DK Aazhivaendhan G Roy R (2022) Treat status assessment of *Chiloschistausneoides* (D.Don) Lindl. (Orchidaceae) as per IUCN criteria. Nelumbo 64(2): 285–289. 10.20324/nelumbo/v64/2022/172596

[B26] POWO (2024) Plants of the World Online. Facilitated by the Royal Botanic Gardens, Kew. Published on the Internet. http://www.plantsoftheworldonline.org/ [Retrieved 15 September 2024]

[B27] Saputra R Mustaqim AM Metusala D Schuiteman A (2020) *Dendrobiumsagin* (Orchidaceae: Epidendroideae), a new species from the Bird’s Head Peninsula, West New Guinea. Phytotaxa 459(2): 190–196. 10.11646/phytotaxa.459.2.9

[B28] Schlechter R (1926) Das system der orchidaceen. Notizblatt des königl. Botanischen gartens und museums zu Berlin 9(88): 563–591. 10.2307/3994326

[B29] Schroth G Laderach P Blackburn CDS Neilson J Bunn C (2015) Winner or loser of climate change? A modeling study of current and future climatic suitability of Arabica coffee in Indonesia. Regional Environmental Change 15(7): 1473–1482. 10.1007/s10113-014-0713-x

[B30] Smith JJ (1905a) Die Orchideen Von Java (Flore de Buitenzorg). EJ Brill Publication, 1–672. 10.5962/bhl.title.86938

[B31] Smith JJ (1905b) Die Orchideen Von Ambon. Landsdrukkerij, 1–125. 10.5962/bhl.title.86926

[B32] Smith JJ (1912) Neue Orchideen Des Malaiischen Archipels. V. Bulletin du Jardin Botanique de Buitenzorg 2(3): 66–68.

[B33] Smith JJ (1939) Die Orchideen Von Java. Bulletin du Jardin Botanique de Buitenzorg 3(16): 133–134.

[B34] Suetsugu K Metusala D Yudistira YR (2021) New Distributional Record of *Lecanorchisnigricans* Honda (Orchidaceae) and a New Addition for the Orchid Flora of Indonesia. Acta Phytotaxonomica et Geobotanica 72(1): 67–71. 10.18942/apg.202010

[B35] Suffan W Metusala D Nisyawati (2021) Micromorphometric analysis of five *Begonia* spp. leaves (Begoniaceae). In: Herdiansyah H et al. (Eds) IOP Conference Series. Earth and Environmental Science 846: 012005. 10.1088/1755-1315/846/1/012005

[B36] Szlachetko DL (2003) Gynostemia Orchidalium III. Orchidaceae - Vandoideae (Polystachyeae, Cymbidieae, Cataseteae, Thecosteleae, Vandeae). Acta Botanica Fennica 176: 1–311.

[B37] Teck OP (2016) A revised description of *Chiloschistasweelimii*, an endemic orchid species from Peninsular Malaysia. Malayan Orchid Review 50: 83–85.

[B38] Trimanto MD Metusala D Erlinawati I Angio MH Yusuf HM Budiarto K (2023) Study of population and conservation of *Dendrobiumcapra* J.J. Smith, an endangered and endemic orchid from Java Island, Indonesia. Journal for Nature Conservation 75: 126476. 10.1016/j.jnc.2023.126476

[B39] Wood JJ (2014) *Chiloschista*. In: Pridgeon AM Cribb PJ Chase MW Rasmussen FN (Eds) Genera Orchidacearum, Volume 6, Epidendroideae 3.Oxford University Press, 1–544.

[B40] Zou LH Huang JX Zhang GQ Liu ZJ Zhuang XY (2015) A molecular phylogeny of Aeridinae (Orchidaceae: Epidendroideae) inferred from multiple nuclear and chloroplast regions. Molecular Phylogenetics and Evolution 85: 247–254. 10.1016/j.ympev.2015.02.014 25725112

